# Drug use among people in prison: A global review of epidemiology, harms and interventions

**DOI:** 10.1111/add.70245

**Published:** 2025-11-20

**Authors:** Louis Favril, John Strang, Seena Fazel

**Affiliations:** ^1^ Department of Criminology, Criminal Law and Social Law Ghent University Ghent Belgium; ^2^ National Addiction Centre, Institute of Psychiatry, Psychology and Neuroscience King's College London London UK; ^3^ Department of Psychiatry Oxford University Oxford UK

**Keywords:** comorbidity, drug dependence, incarceration, injecting drug use, jail, overdose, recidivism

## Abstract

**Background and aims:**

People who use drugs are overrepresented in the criminal justice system. We aimed to provide a broad synthesis of the epidemiology, harms and interventions related to drug use and drug use disorders among incarcerated adults worldwide, and highlight gaps in evidence and practice.

**Methods:**

In this structured review, we searched PubMed and Web of Science for articles published between 2015 and August 2025. Systematic reviews, meta‐analyses and large primary studies with unselected samples were given preference for inclusion.

**Results:**

Four in ten adults who enter prison meet diagnostic criteria for a drug use disorder, a treatable psychiatric condition often underlying the offences that led to their incarceration. Drug use disorders are approximately ten times more prevalent among people in prison compared with the general population, with a higher excess in women. Comorbid mental disorders are common. Around a third of incarcerated individuals report using drugs during imprisonment, which poses a health and safety risk for people both living and working in prisons. Injecting drug use in prison contributes to blood‐borne virus transmission. In addition to its strong link with recidivism, drug use is associated with a markedly increased risk of premature mortality after release from prison, particularly from drug‐related causes within the first two weeks post‐release. Despite robust evidence supporting the effectiveness of prison‐based pharmacological (e.g. opioid agonist treatment) and psychosocial (e.g. therapeutic communities) interventions in reducing drug‐related harms, there remains a significant treatment gap within prison settings worldwide. Further research is needed to assess the health benefits of harm reduction services in prisons, including needle and syringe programmes. Strategies to facilitate linkage to and retention in post‐release services are key to ensure continuity of care and achieve sustainable treatment outcomes.

**Conclusion:**

The high prevalence of drug use and its multiple adverse outcomes among people in prison underscore the need for provision of evidence‐based interventions. Expanding and integrating prison‐based and post‐release interventions to address drug use has the potential to yield both public health and criminal justice benefits.

## INTRODUCTION

Drug use, which includes the use of illicit drugs (e.g. cannabis, cocaine, amphetamines and heroin) and extramedical use of prescription drugs (e.g. opioid analgesics and benzodiazepines), is widespread throughout the world. In 2023, an estimated 316 million people, or 6% of the global population aged 15 to 64 years, used drugs in the past year [1]. Drug use occurs along a continuum of severity [2]. For most people, drug use will remain infrequent and transient, with minimal harms. However, of the estimated 25% of people worldwide who have used drugs in their lifetime, approximately one in seven (14%) will develop a drug use disorder at some point [3]. In this review, we use the term drug use disorder to refer to a harmful pattern of use associated with clinically significant impairment and distress, consistent with diagnostic criteria outlined in the Diagnostic and Statistical Manual of Mental Disorders, Fifth Edition, and International Classification of Diseases 11th Revision [2]. In the literature, this term is often used interchangeably with drug dependence and addiction, even though definitions differ.

A cross‐national analysis of epidemiological surveys from 25 countries spanning various regions and income levels estimated the prevalence of drug use disorders in the general adult population at 0.7% within the past year and 3.5% over the lifetime [3]. Drug use disorders contribute substantially to the global burden of disease [4, 5] owing to their early onset, chronic or relapsing course, long time to remission [2] and poor health outcomes. People who experience drug use disorders are at increased risk of mental and physical health morbidity, disability and pre‐mature mortality [6, 7, 8, 9, 10, 11]. In addition, drug use disorders impose a substantial burden on families, social networks and society as a whole through adverse impacts on socio‐economic and criminal justice domains [1, 12, 13].

Extensive research has documented a strong association between drug use and criminal offending [14, 15, 16, 17, 18, 19], although the nature of this relationship is complex [20]. There are various pathways from drug use to crime, including engagement in acquisitive crimes to financially support addiction, criminal behaviour (especially violence perpetration) driven by the psychopharmacological effects of drug use, and the illegal nature of drug markets promoting opportunities for involvement with criminal networks [20]. At a broader level, determinants of social exclusion (e.g. poor educational attainment, unemployment, unstable housing and poverty) increase vulnerability to both drug use and offending [21, 22, 23]. In addition, punitive drug laws and policies that criminalise drug use tend to funnel people who use drugs into the criminal justice system [24]. The consequence of these multiple intersecting factors is that prisons around the world detain large numbers of individuals who use drugs. With over 30 million people transitioning through prisons annually [25], addressing drug use in this population has the potential to improve public health and safety [26, 27].

## AIMS AND METHODS

In this structured review [28], we provide a broad synthesis of the epidemiology, harms and interventions related to drug use and drug use disorders among incarcerated adults worldwide, and highlight gaps in evidence and practice. Although also highly prevalent in prison populations, licit substances such as tobacco [29] and alcohol [30] are beyond the scope of this review. The focus is on incarcerated adults and we do not discuss drug use in juvenile offenders [31], people serving community sentences [32] and those residing in other detention settings such as police custody, secure (forensic) psychiatric hospitals and immigration detention centres. Issues pertaining to the supply of drugs in prisons (e.g. smuggling routes and drug markets) [33, 34] are also not addressed.

We searched PubMed and Web of Science for articles published between 2015 and 31 August 2025. Search terms included (prison* OR jail* OR incarcerat*) and (drug* OR substance*), combined with specific keywords relating to epidemiology, harms and interventions. Additional articles were identified by scanning reference lists of relevant publications. Papers were selected based on topical relevance and methodological quality, with final decisions reached through consensus. To minimise bias and ensure robust conclusions, systematic reviews, meta‐analyses and large primary studies with unselected samples [35] were given preference for inclusion. Primary studies, particularly those not covered in systematic reviews, were selected to highlight key findings. Qualitative research was included to complement quantitative evidence. Although the focus was on evidence published within the past decade (2015–2025), we supplemented this literature with highly cited older studies. Overall, the current literature review was primary based on 56 key publications, of which 34 were review articles and 22 primary studies (see Data S1), with additional publications cited throughout the paper. Because of the wide variation in research designs and outcomes, a structured narrative approach was chosen to synthesise findings, organising evidence by key thematic areas—epidemiology, harms and interventions. The analysis was not pre‐registered and results should be considered exploratory.

Although in the United States (US) there is a clear distinction between jails (local facilities holding people awaiting trial or serving sentences typically less than 1 year) and prisons (state and federal facilities detaining people serving longer sentences), most other countries do not make this distinction. Therefore, as this review has an international scope, we use the term prisons to refer to all correctional facilities [26].

## EPIDEMIOLOGY

### Patterns of drug use

When interpreting epidemiological evidence on drug use among people in prison, regardless of whether they meet diagnostic criteria for a drug use disorder, there are two important considerations. First, most studies rely on self‐report methods (e.g. surveys) to assess drug use [36, 37, 38, 39], which are susceptible to social desirability and recall bias [40]. However, a recent meta‐analysis [41] found good agreement between self‐report and objective measures (biological testing) of drug use, including in criminal justice populations. Second, available prevalence data are patchy owing to methodological differences in sampling and measurement [36, 37, 38]. For example, studies vary in the number and types of substances assessed, the frequency of use (e.g. at least once vs. daily) and timeframe considered (e.g. lifetime vs. past‐month). Although this heterogeneity makes it challenging to draw clear conclusions about the patterns of drug use in prison populations, there are several consistent trends in the research literature.

First is the high prevalence of drug use *before* imprisonment. A 2022 systematic review of 26 European studies [36] documented that 61% of people in prison have a history of drug use before imprisonment (Table 1). In a national US survey of nearly 25 000 incarcerated individuals, 64% reported using drugs in the 30 days before their arrest and 38% did so at the time of their offence [44]. Regarding frequency of use, almost half (47%) of people in Norwegian prisons used drugs on a daily basis during the 6 months preceding their incarceration [45]. Overall, cannabis, cocaine and amphetamines are the drugs most commonly used before imprisonment, often in combination with other substances [36, 44, 45, 46]. Despite geographical differences [47], people in prison consistently report much higher rates of drug use before imprisonment than found in the general population [48, 49].

**TABLE 1:** Prevalence of drug use, drug use disorders and comorbidity among people in prison.

	Prevalence (%)	Region
Drug use		
Before imprisonment [36]	61	Europe
During imprisonment [39]	32	Global
Drug use disorders		
On reception to prison [26]	39	HIC
Lifetime prevalence [42]	31	LMIC
Comorbidity^a^		
Current prevalence [43]	16	Global
Lifetime prevalence [43]	30	Global

Abbreviations: HIC, high‐income countries; LMIC, low‐income and middle‐income countries.

^a^ Co‐occurring drug use disorder and mental disorder.

Second, although institutional policies prohibit drugs from entering prisons, drug use is common *during* imprisonment [37]. In low‐income and middle‐income countries (LMIC) specifically, a 2018 meta‐analysis pooling data from 26 samples estimated that 25% of people in prison used drugs during imprisonment [47]. Based on findings from 81 studies published up to 2022, a global review [39] concluded that the prevalence of in‐prison drug use was 32% across 22 countries (Table 1). Similarly, recent large‐scale surveys of unselected samples in Norway [45] and Belgium [46] found that approximately a third (35%) of people in prison used drugs while incarcerated, most commonly cannabis, benzodiazepines and opioids. The preference for these particular drugs corresponds with the main reasons why people use drugs in prison, namely to relieve stress, forget problems and counteract boredom [38, 50], suggesting that drug use may serve as a means to cope with the challenges of imprisonment [38]. However, the types of drugs used in prison also tend to mirror those prevalent in the surrounding community, such as cocaine in Latin America and heroin in South Asia [39]. The use of new psychoactive substances (NPS) in prisons is increasingly reported in several countries [51, 52], particularly synthetic cannabinoids in the United Kingdom (UK) [53, 54], although availability of these drugs is influenced by national legislation and regulation [55]. Regarding the route of administration, the prevalence of injecting drug use while incarcerated has been estimated at 2% in LMIC [47], although global estimates are currently lacking. Overall, sex differences in drug use during imprisonment are not consistent across primary studies [38]. Pre‐prison drug use is among the strongest predictors of in‐prison drug use [38], associated with a sevenfold increased risk [45, 46].

Third, research has highlighted the impact of incarceration on drug use patterns [46, 56, 57, 58, 59, 60, 61, 62]. Comparative analyses show that the prevalence of drug use during imprisonment is consistently lower than before imprisonment [46, 47, 49], suggesting that incarceration contributes to a reduction in use. Indeed, recent representative prison surveys in high‐income countries (HIC) indicate that 50% to 60% of people who used drugs before their incarceration cease doing so once incarcerated [46, 56]. Moreover, among those who do continue to use drugs in prison, both the quantity of drugs used and frequency of use typically decrease during imprisonment relative to pre‐incarceration levels of use [39, 59]. Although restricted availability and high cost of drugs in prisons influence drug use patterns [39, 60], there is little evidence to suggest that only supply factors account for this reduction in drug use. In a survey of 800 people in French prisons, for example, personal choices were frequently cited as reasons for discontinuing drug use during imprisonment [61]. Qualitative research inquiring into the experiences of drug‐involved women supports this, in that incarceration can provide a (temporary) respite from their lives in the community that were characterised by addiction [63, 64].

The impact of incarceration is further demonstrated by a change in types of drugs used. Research shows a consistent trend that people who used stimulants (e.g. cocaine and amphetamines) before imprisonment switch to using depressants (e.g. opioids and benzodiazepines) during imprisonment [46, 56, 57, 58, 59]. One possible explanation for this shift may be that some drugs are less readily available in prison than others. Alternatively, it could be argued that the psychopharmacological effects experienced from depressants are more compatible with a stressful prison environment than those of stimulants [38]. Further research is needed to better understand how supply and demand dynamics influence drug‐related choices during imprisonment.

Incarceration can also contribute to more harmful patterns of drug use in several ways [38, 60], although evidence in this area is less strong. First, mandatory drug testing may incentivise people in prison to switch from using drugs that can be traced long after use (such as cannabis) to those with shorter excretion times (such as opioids), resulting in potentially increased harm [65]. Second, people may adopt a more harmful pattern of use in prison by altering their route of administration, from smoking or ingestion to injecting, to maximise the efficiency of expensive drugs that are limited in supply [60]. Third, evidence shows that people who continue to inject drugs once incarcerated engage in injecting risk behaviours (e.g. needle and syringe sharing) more frequently than they did before imprisonment [66], likely because of limited availability of sterile injecting equipment within prisons [60, 67]. Fourth, drug use may be initiated in prison [62]. Although estimates vary widely owing to methodological differences between studies, approximately 3% to 10% of people in prison report that they have first started using drugs while incarcerated [37, 45]. The stressful nature of incarceration, along with exposure to drugs and peer pressure [68], may contribute to initiation of drug use and trigger relapse after a period of abstinence. This contrasts with the broader trend described earlier, in which drug use tends to decline during imprisonment, highlighting the complexity of the prison setting.

However, drug use does not equate to the presence of a drug use disorder. The proportion of incarcerated individuals whose use of drugs meets the clinical threshold for a drug use disorder is not well understood. Some smaller studies suggest that more than half (53%–60%) of people in prison in England [69] and Norway [70] who reported using drugs screen positive for drug dependence. Although many people in prison appear to use drugs at levels that do not necessarily require treatment, they remain at risk of developing a drug use disorder.

### Drug use disorders

Drug use disorders are among the most prevalent psychiatric disorders within the prison population [26, 71]. In a meta‐analysis pooling diagnostic interview data from 24 studies in HIC [72], four in 10 (39%) adults met diagnostic criteria for a drug use disorder on reception to prison (Table 1) [26], with meta‐regression indicating that rates have increased over time. Another meta‐analysis of 11 non‐admission samples from LMIC estimated the 12‐month and lifetime prevalence of drug use disorders at 5% and 31%, respectively [42]. In a multi‐national cohort study based on patient registry data, about one‐third (27%–36%) of people in Nordic prisons had a diagnosed drug use disorder during the period spanning 2010 to 2019 [73]. The most prevalent drug use disorders in the latter study involved cannabis, opioids and stimulants, with polysubstance use being common [73].

Three overarching findings are noteworthy in relation to drug use disorders. First, there is a higher prevalence in admission studies (on entry to prison) compared with cross‐sectional investigations that include people at later stages of imprisonment [42, 43], which is consistent with findings on drug use patterns (as described above) and longitudinal studies reporting higher rates of psychiatric morbidity at intake to prison [74]. Prevalence on reception to prison provides information about drug use disorders before imprisonment. In turn, estimates of current prevalence (based on non‐admission samples) may better represent the average disease burden during imprisonment, for which global estimates are currently not available [26]. Equally lacking are incidence data on drug use disorders in prison and their course during imprisonment has yet to be examined in prospective cohort studies. Second, research has consistently found higher rates of drug use disorders among incarcerated women than men [44, 72, 73], while the opposite pattern is observed in the general population [3, 4]. For example, on reception to prison, 51% of women meet diagnostic criteria for a drug use disorder compared to 30% of men [72]. This sex difference could be explained by much lower rates of female incarceration [75], and hence, women in prison (who typically constitute <10% of the prison population) representing a more selected group of high‐risk individuals characterised by elevated levels of social vulnerability, mental illness and drug use [76, 77, 78]. Third, rates of drug use disorders are substantially higher among people in prison compared with the general population [42, 48, 73, 79, 80]. Pooled prevalence ratios in LMIC indicate that drug use disorders are six times more common among people in prison relative to their community peers [42]. In Nordic countries, the prevalence of drug use disorders at prison intake is more than 10 times higher compared to the general population [73], with similar findings (12 times higher) found in the United States [80]. This excess prevalence is most pronounced for opioid use disorder relative to other drug use disorders [48, 79].

### Psychiatric comorbidity

People in prison bear a disproportionate burden of psychiatric morbidity, including high rates of psychosis, depression, post‐traumatic stress disorder, attention‐deficit hyperactivity disorder and personality disorders [26]. Poor mental health is a replicated risk factor for in‐prison drug use [38] and many studies have highlighted a substantial overlap between mental disorders and drug use disorders in prison populations worldwide [43, 48, 81, 82, 83]. For example, a 2022 meta‐analysis of 50 studies from 21 countries found that a third of incarcerated individuals with current depression (34%) and psychosis (37%) have a comorbid drug use disorder [43]. In the total prison population, the current and lifetime prevalence of any co‐occurring mental disorder (other than personality disorders) and drug use disorder (also termed dual disorders) is estimated at 16% and 30%, respectively (Table 1) [43]. Other meta‐analyses have reported a two times higher likelihood of substance use disorders (including alcohol) among people in prison with post‐traumatic stress disorder [84] and attention‐deficit hyperactivity disorder [85]. Overall, psychiatric comorbidity is more prevalent among incarcerated women than men [81, 82, 86].

The mechanisms underlying dual disorders are complex and difficult to disentangle as these disorders interact [87]. First, symptoms of mental disorders, such as impulsivity and mood dysregulation, may increase risk of developing a drug use disorder, and people may use drugs as a means of self‐medication to alleviate mental health symptoms. Second, drug use may also contribute to the onset and persistence of mental disorders, including substance‐induced conditions. Third, mutual underlying causes (e.g. genetic vulnerabilities and adverse childhood experiences) may further account for the high rates of comorbid mental disorders among people with drug use disorders. Regarding temporal order, epidemiological surveys in the general population indicate that the onset of mental disorders typically precedes that of drug use disorders [3, 88]. In any case, having a co‐occurring mental disorder can worsen the prognosis and increase the severity of drug use disorders, which is associated with poorer treatment outcomes [87].

## HARMS

### During imprisonment

Drug use poses a health and safety risk for people both living and working in prisons. The presence of drugs in prisons can impact on violence and contribute to debt and the illicit prison economy, undermining prison security and negatively affecting the social climate within prisons [89, 90, 91, 92]. Drug use disorders are further associated with suicidal behaviour [93], with a 2022 meta‐analysis [94] reporting a twofold increased odds [odds ratio (OR) = 2.23, 95% confidence interval (CI) = 1.51–3.28] of suicide attempts during imprisonment. In two case–control studies, 70% of men [95] and 45% of women [96] who engaged in near‐lethal self‐harm while incarcerated met diagnostic criteria for a current drug use disorder. Withdrawal from drugs is a possible trigger for suicide during the early period of incarceration [97, 98]. In recent years, NPS (and synthetic cannabinoids in particular) have been implicated in over half (57%) of all non‐natural deaths in English and Welsh prisons [99]. Because NPS are typically many times more potent than other drugs, their use may come with a heightened risk of overdose. More research is needed to assess the health and behavioural outcomes associated with the use of NPS in prisons. Additionally, injecting drug use carries particular risks. In prisons, health consequences associated with injecting drug use include a much greater likelihood of exposure to blood‐borne viruses as a result of sharing contaminated injection paraphernalia [60, 66]. Overcrowding and poor living conditions in prisons further create high‐risk environments that drive infectious disease transmission [100]. A meta‐analysis [101] covering data published between 2005 and 2015 found that incarcerated individuals who inject drugs have six times the prevalence of HIV [prevalence ratio (PR) = 6.0, 95% CI = 3.8–9.4], eight times the prevalence of hepatitis C virus (PR = 8.1, 95% CI = 6.4–10.4), and two times the prevalence of hepatitis B virus (PR = 2.0, 95% CI = 1.5–2.7) compared with their non‐injecting peers.

### After imprisonment

The vast majority of incarcerated people will eventually return to the community. Although incarceration may provide a period of abstinence or reduced drug use, this decrease in use is often temporary, with many released individuals resuming drug use at levels similar to those before imprisonment [102, 103, 104]. People released from prison typically return to environments characterised by drug use, poor living conditions, significant social and economic challenges and limited access to services–all of which may trigger relapse to drug use [104, 105, 106]. Drug use is associated with substantial health and societal harms post‐release, particularly premature mortality and recidivism.

Formerly incarcerated individuals face a markedly increased risk of premature mortality [107, 108, 109, 110, 111]. A 2024 meta‐analysis pooling data from 1.5 million people in mostly HIC showed that this excess mortality is largely attributable to drug‐related causes in the early period following release [111]. In a seminal study of more than 30 000 individuals released from Washington State prisons, the risk of death from drug overdose during the first 2 weeks post‐release was over 100 times higher compared to the general population [112]. Internationally, drug overdose is the leading cause of death after release from prison, accounting for 76% of all deaths within 2 weeks of release and 59% within 3 months [107]. Opioids are involved in nearly 60% of post‐release overdose deaths in the United States [113, 114, 115]. Factors that may contribute to this high risk of overdose mortality shortly after prison release include a decreased physiological tolerance resulting from a period of abstinence or reduced drug use during imprisonment [105, 116], increased risk‐taking behaviour on release and the concurrent use of multiple substances [117]. Overdoses may also be intentional [105]. A nationwide cohort study in Sweden [118] found that the association between drug use disorders and all‐cause mortality after release [hazard ratio (HR) = 1.67, 95% CI = 1.53–1.83] is independent from the contribution of socio‐demographic, criminological and familial factors, supporting a possible causal effect. Non‐fatal drug overdoses are also common during the post‐release period [119, 120], resulting in considerable morbidity.

In addition to individual health consequences, drug use among people released from prison has a large societal impact through its association with recidivism. Many people who use drugs repeatedly cycle in and out of prison. Controlling for socio‐demographic and criminological factors, a retrospective cohort study of nearly 50 000 people released from Swedish prisons [121] documented a significant relationship between drug use disorders and violent reoffending in both men (HR = 1.65, 95% CI = 1.58–1.72) and women (HR = 1.84, 95% CI = 1.46–2.30). Prospective cohort studies in other HIC found that individuals who screened positive for pre‐prison drug dependence (HR = 4.20, 95% CI = 2.95–5.97) [122] and those who injected drugs after release from prison (HR = 2.04, 95% CI = 1.60–2.61) [123] are more likely to be re‐incarcerated than their peers without these risk factors. Overall, risk of recidivism is higher in people released from prison with dual disorders compared to those with either a substance use disorder or mental disorder alone [121, 124, 125, 126, 127].

## INTERVENTIONS

The high prevalence of drug use and its multiple adverse outcomes among people in prison underscore the need for provision of evidence‐based interventions. Incarceration provides a unique opportunity to identify unmet needs and initiate treatment for a vulnerable group of individuals who are otherwise hard to reach by health and social services in the community [26]. In addition to screening, a variety of interventions can be offered in prisons, which differ in approach but share the aims of reducing drug use, drug‐related offending and associated health problems. Overall, the robustness of evidence for prison‐based interventions varies widely, with key limitations including selection bias and high attrition rates [128, 129, 130]. Further, heterogeneity in study design, samples and outcome measures make a synthesis of the literature challenging. In terms of research gaps, a 2024 systematic review [130] of 126 studies evaluating the effectiveness of a broad range of interventions for drug use in prison populations found that most research was conducted in HIC (96%) and examined post‐release outcomes (90%), highlighting the need for more data from LMIC and greater research attention to outcomes while individuals are still incarcerated.

### Screening

Screening on reception to prison allows for the early identification of drug use disorders and related needs, enabling timely and appropriate treatment planning. In a recent study of incarcerated men in Norway [70], the Drug Use Disorder Identification Test (DUDIT) was found to perform well [area under the receiver operating characteristic curve (AUROC) >0.9] in detecting harmful patterns of drug use, with a five‐item version of the DUDIT showing high sensitivity (97%) and specificity (83%). The Alcohol, Smoking and Substance Involvement Screening Test (ASSIST) is another brief screening tool, developed by the World Health Organization, demonstrating good accuracy (AUROC range = 0.7–0.9) in predicting drug use after release from prison [131]. However, to date, no systematic reviews have assessed the predictive performance of screening instruments for drug use in prison populations. Available evidence indicates that screening, brief intervention and referral to treatment (SBIRT) programmes in prisons do not have clear impacts on drug use, criminal and health outcomes [130, 132].

### Psychosocial treatments

In general, most psychosocial treatments delivered in prisons have a weak evidence base in support, with most research coming from the United States [128, 129, 130]. Recent systematic reviews indicate that cognitive behavioural therapy, as a standalone intervention, may not be effective in reducing drug use and recidivism (Table 2) [128, 129, 130], although design limitations prevent clear generalisability. Research on self‐help interventions (including 12‐step mutual aid groups, such as Narcotics Anonymous, and other peer‐based support programmes) in prison settings is scarce and currently does not support their effectiveness [128, 129, 130]. By contrast, there is compelling evidence that therapeutic communities reduce recidivism and, to a lesser extent, drug use after release [128, 129, 130, 137, 138]. For example, a 2024 meta‐analysis [130] based on cohort studies with data linkage showed that therapeutic communities reduced re‐arrest at 6 to 12 months after release (OR = 0.72, 95% CI = 0.59–0.87) and re‐incarceration at 24 months (OR = 0.68, 95% CI = 0.48–0.96). Prison‐based therapeutic communities, which are typically located in a separate prison wing or facility, may be more effective than other psychosocial treatments because of their structured, intensive and long‐term approach to addressing the multiple factors underlying drug use. Taken collectively, the wide variability within psychosocial treatments makes it challenging to draw definitive conclusions about their overall effectiveness.

**TABLE 2:** Effectiveness of prison‐based interventions addressing drug use.

Intervention	Evidence
*Psychosocial interventions*	
Cognitive behavioural treatment	Mixed findings; generally not effective as a standalone intervention in reducing drug use and recidivism [128, 129]
Self‐help interventions	Limited research providing little evidence to support their effectiveness [129, 130]
Therapeutic communities	Largely consistent evidence for reducing recidivism and, to a lesser extent, drug use after release [128, 129, 130]
*Pharmacological treatments*	
Opioid agonist treatment	Consistent evidence for increasing community treatment engagement and reducing mortality and drug use, whereas no significant impact on recidivism [130, 133]
Extended‐release buprenorphine	Growing evidence for reducing opioid use and improving treatment retention post‐release [134]
Naltrexone	Limited evidence with mixed findings [133, 134]
*Harm reduction interventions*	
Needle and syringe programmes	Limited and low‐quality evidence; suggestive of health benefits by preventing injection‐related infections [130, 135]
Take‐home naloxone	Potential reduction in post‐release overdose deaths but insufficient evidence to draw reliable conclusions [130, 136]

### Pharmacological treatments

Opioid agonist treatment (OAT) with methadone or buprenorphine maintenance is a highly effective treatment for opioid use disorder [9, 139] and robust evidence indicates that OAT in prison settings is associated with a wide range of positive health and treatment outcomes (Table 2) [128, 130, 133, 136, 140]. For example, a 2019 meta‐analysis [133] pooling trial data from HIC concluded that methadone provided during imprisonment reduces illicit opioid use (OR = 0.22, 95% CI = 0.15–0.32) and injecting drug use (OR = 0.26, 95% CI = 0.12–0.56) after release, as well as increasing community treatment engagement (OR = 8.69, 95% CI = 2.46–30.75). Other meta‐analyses found OAT to reduce all‐cause [relative risk (RR) = 0.24, 95% CI = 0.17–0.35] and drug‐related (RR = 0.20, 95% CI = 0.12–0.34) mortality in the first 4 weeks after release from prison [130, 141], while there is no clear effect of OAT on recidivism outcomes [130, 133]. Evidence on the relative benefits of different OAT options in prison settings is limited [133, 136]. Extended‐release buprenorphine (administered by injection on a weekly or monthly basis) is an emerging treatment option [139] and two narrative reviews [134, 142] have recently described its feasibility, safety and potential effectiveness (in reducing opioid use and improving treatment retention) in prison populations. In particular, when administered at the point of release, these long‐acting formulations can serve as a transitional bridge to reduce the risk of post‐release overdose and increase retention in community treatment by overcoming the need for daily dosing [142]. In contrast to OAT, prison research on the effects of naltrexone, an opioid antagonist, is relatively scarce and generally shows inconsistent findings [130, 133, 134, 136]. Overall, as these pharmacological interventions are suitable only in the context of opioid use, people in prison who use non‐opioid drugs remain poorly served by current pharmacotherapies [10].

### Treatment coverage

Many people in prison experience a need for treatment related to drug use [143, 144, 145]. However, research indicates that a substantial proportion of them do not receive the treatment they need [145, 146, 147]. For example, a nationally representative US study found that only about a quarter (22%–28%) of people with a drug use disorder had received any treatment since admission to prison [80]. Higher rates have been reported in Norway, a country with universal health coverage, where two‐thirds (64%) of people who screened positive for drug dependence received specialised treatment in prison [148]. Overall, in HIC, foreign nationals and those with a shorter duration of incarceration are less likely to receive prison‐based treatment [146, 147, 148], whereas polydrug use is positively associated with receiving treatment [147, 148]. However, it remains unclear whether this treatment gap reflects limited service availability, restricted accessibility or low uptake. The unmet need for treatment is likely even greater in LMIC because of resource constraints [42], although reliable data to support this are currently lacking.

Specifically regarding pharmacological treatments, the global coverage of OAT in prisons remains low [149]. According to a scoping review, key reasons cited by professional stakeholders for not providing OAT in prison include security and diversion concerns, negative attitudes toward OAT and a preference for abstinence‐oriented treatments [150]. Regulatory, logistical and financial factors also have a role. Prison authorities may choose to discontinue OAT for patients when they enter prison, and forced withdrawal from OAT in prison has been shown to reduce the likelihood of people re‐engaging in OAT after release [151, 152]. Importantly, availability of OAT in prison does not equate to easy access [153]. Qualitative research shows that people experience multiple challenges to accessing OAT during imprisonment, including treatment waitlists and restrictive policies [154, 155]. For example, in US jails where OAT is available, access is often restricted to specific groups such as pregnant individuals and those already receiving OAT before incarceration [156].

Taken together, these findings highlight a significant treatment gap in prisons. Yet, international standards assert that people in prison should have access to the same standards of health care available in the community [157, 158]. Improving the availability and accessibility of evidence‐based treatments within prison settings is essential to realising this principle of equivalence, which will require structural funding and political commitment.

### Harm reduction

Harm reduction interventions, which seek to reduce harms caused by ongoing drug use, include needle and syringe programmes (NSP) and take‐home naloxone (THN).

By providing sterile equipment to people who inject drugs in prison, NSP primarily aim to prevent transmission of blood‐borne viruses. Prison‐based NSP are currently available in only nine countries worldwide [159]. Resistance toward implementation of NSP in prisons centres mostly on workplace health and safety concerns by staff and on whether such services will result in encouraging drug use [160, 161]. Although the health benefits of NSP have been documented in community settings [162], evidence to support their effectiveness in prisons remains scarce [130]. A 2018 systematic review [135] identified only five studies assessing the health outcomes of NSP in prison settings and, although the overall methodological quality was rated as low, findings were suggestive of health benefits by preventing infectious diseases (Table 2), with minimal negative consequences observed. Recently, two cost–benefit analyses indicated that expanding NSP provision in Canadian prisons [163] and introducing NSP in Australian prisons [164] would result in substantial health care savings related to hepatitis C virus and other injection‐related infections. Specifically, each dollar spent on prison‐based NSP was estimated to save between $2 and $2.6 in treatment costs [163, 164]. However, confidentiality concerns have been identified as an important barrier to service uptake [165]. For example, fear of being discriminated against by prison authorities deterred people from participating in a pilot NSP in Portuguese prisons, ultimately resulting in the programme's termination [160].

The administration of naloxone, a short‐acting opioid antagonist, is an effective bystander intervention for overdose reversal [166]. THN programmes, which provide naloxone distribution alongside overdose management training to people with (a history of) opioid use disorder at the point of release, have the potential to reduce opioid‐related overdose deaths in people released and their peers [167], although limited research has assessed its effectiveness in prison populations (Table 2) [130, 136]. An evaluation of a national prison‐based THN programme in Scotland using observational data reported a continuous decrease in opioid overdose mortality within the 4 weeks after release [168, 169]. Although studies indicate widespread support for THN from both people in prison and professional stakeholders [170, 171], their implementation in prison settings is faced with significant barriers and challenges [172], with only 11 countries currently providing this intervention to people being released [149]. Overall, training and preparation of willing and capable bystanders at an overdose event are key factors to realise the potential of this intervention [173].

### Challenges

Although prisons provide a setting that removes some of the barriers to treatment access that incarcerated individuals may have encountered in the community, they also present distinct challenges to delivery of treatment [174]. Prisons, where security is typically prioritised over care, are not tailored to function as therapeutic environments for addressing drug‐related issues. Frequent movements between prisons (often with no transfer of medical records) and short durations of incarceration make sustained engagement in treatment difficult, and structural barriers such as overcrowding and understaffing further impede the optimal delivery of care [26]. At the individual level, barriers including distrust of the health care system, low health literacy and help‐seeking behaviour, and fear of stigmatisation can prevent people in prison from accessing available services [175]. Moreover, the widespread availability of drugs in prisons can reduce motivation to seek treatment and undermine efforts to achieve abstinence by those in treatment. These challenges are exacerbated by a broader prison culture in which drug use is accepted, normalised and often pressurised [38].

Relatedly, the specificity of the prison context also poses logistical challenges for conducting methodologically rigorous intervention studies [176]. Prison researchers encounter many obstacles, including difficulties with follow‐up (e.g. because of the rapid turnover of prison populations) and institutional constraints on scheduling and implementation of trials [177]. More research‐friendly prisons will be required to improve the evidence base of interventions to reduce harms related to drug use.

### Continuity of care

Although prison‐based interventions are essential, any gains made during imprisonment may be lost if support is not available on release into the community. Considering the high relapse rates, strategies to facilitate linkage to and retention in post‐release services, including discharge planning and transitional care coordination, are key to ensure continuity of care and achieve sustainable treatment outcomes [26]. Structured, simple and scalable assessment tools to identify those at highest risk may assist in this process [178]. Achieving throughcare may be more seamless in countries where prison and community health services are overseen by the same governing body [179]. However, few interventions aimed at facilitating continuity of care for people with drug‐related treatment needs during their transition from prison to the community have been rigorously evaluated to date, with inconsistent results [180, 181, 182]. Currently, there is little evidence to support the effectiveness of case management in reducing recidivism or drug use after release from prison [130]. High rates of treatment attrition and lack of sustained benefits post‐release indicate that more intensive and longer‐duration interventions may be needed [182]. Emerging evidence suggests that peer‐led services, in which individuals with lived experience of incarceration provide support and guidance to those released from prison [183], may be a promising community‐based approach to improve treatment adherence [184]. More broadly, strategies should focus on reducing the structural barriers to treatment engagement that people are commonly faced with on release, including housing instability and lack of employment [185, 186].

### Diversion

Addressing drug use requires a public health framework rather than a criminal justice approach [24]. As incarceration may not be an effective response to drug use, models to link offenders who use drugs to community‐based treatment, as an alternative to incarceration, have been implemented at different stages of the criminal justice system [187, 188, 189]. Drug courts are one common example of such diversion schemes [190]. Instead of punishment, their purpose is to address drug use as the underlying driver of criminal behaviour by incentivising individuals with a drug use disorder to enter treatment with an agreement that charges against them will be reduced or dismissed on treatment completion. Evaluation studies of drug courts are often confounded by participant selection bias. Using quasi‐experimental designs, a recent prospective cohort study based on nationwide registry data demonstrated that drug courts in Sweden effectively reduce substance use, crime and adverse health outcomes [191]. Within‐individual analyses further suggested that the same individual had longer times‐to‐event for all outcomes during the period when they were in drug court treatment compared with periods on parole or probation [191]. In the United States, similar diversion schemes for low‐level drug offenders were found to be cost‐effective, generating savings in the criminal justice system while only moderately increasing health care costs [192]. Looking further upstream, police‐based diversion programmes have shown promising results in preventing future contact with the criminal justice system and drug‐related harms [193], although these findings require replication in high‐quality trials.

## CONCLUSION

In this review of research published in the past decade, we synthesised evidence on the epidemiology, harms and interventions related to drug use and drug use disorders among incarcerated individuals worldwide, and outlined a number of research recommendations (Box 1). Four in 10 adults who enter prison meet diagnostic criteria for a drug use disorder and approximately a third of people in prison report using drugs during imprisonment. Incarceration provides an opportunity for identifying and treating people with harmful patterns of drug use who are otherwise difficult to reach by services in the community. However, this potential is typically unrealised owing to limited resources and poor access to evidence‐based interventions. After release from prison, people who use drugs are at increased risk of premature mortality and reoffending, such that effective responses to drug use in prison populations are likely to yield benefits for both health and criminal justice systems.

Box 1 **Key research recommendations**

**Epidemiology**

- Future prison surveys of prevalence and needs should use standardised measures of drug use to allow for comparability across settings.
- More research is needed to better understand how supply and demand dynamics influence patterns of drug use during imprisonment.
- Longitudinal studies are required to clarify the incidence and course of drug use disorders before, during and after imprisonment.

**Harms**

- Further investigation is required to assess the health and behavioural outcomes associated with the use of NPS in prison.
- Epidemiological research should examine the rates, causes and risk factors for mortality after release from incarceration in LMIC.
- Risk assessment and modelling tools, leading to risk stratification, for post‐release harms should be developed and validated.

**Interventions**

- In LMIC, treatment effectiveness of prison‐based psychosocial and pharmacological interventions to reduce harms related to drug use should be evaluated.
- More evidence is required to determine whether self‐help interventions and mutual aid groups in prisons are effective in addressing drug use.
- Trials should assess the relative benefits of different OAT options in prisons, with special attention to extended‐release (injectable depot) buprenorphine.
- Studies should shift from assessing acceptability and feasibility toward evaluating the effectiveness of NSP and THN programmes in prison settings.
- Clarification is needed for the most effective approaches to managing dual disorders among people in prison, including integrated treatment models.
- Further research is required to identify effective strategies that facilitate linkage to and retention in post‐release services to improve continuity of care.

Abbreviations: LMIC, low‐income and middle‐income countries; NPS, new psychoactive substances; NSP, needle and syringe programmes; OAT, opioid agonist treatment; THN, take‐home naloxone.

## AUTHOR CONTRIBUTIONS

**Louis Favril:** Conceptualization (lead); formal analysis (equal); methodology (equal); writing—original draft (lead); writing—review and editing (equal). **John Strang:** Formal analysis (equal); writing—original draft (equal); writing—review and editing (equal). **Seena Fazel:** Formal analysis (equal); methodology (equal); writing—original draft (equal); writing—review and editing (equal).

## DECLARATION OF INTERESTS

L.F. and S.F. declare that they have no competing interests. J.S. is a clinician and academic who has worked with local, national and international government and non‐government agencies to develop and test new approaches to tackling addiction and related problems. Through his employer (King's College London), he has received research and project grant support from a range of government and charitable research agencies and charitable organisations. Through the university, he has also worked with pharmaceutical and technology companies from some of whom the university has received project grant support and/or honoraria and/or consultancy payments, as described at https://www.kcl.ac.uk/people/john-strang (including, in the past 3 years, MundiPharma, Camurus, Pneumowave, Accord and dne) and also medication or device supply (Pneumowave, CMI and Catalent) to develop or study potentially improved formulations and devices. J.S. is also named as a Patron of Addiction Family Support (formerly DrugFAM), a UK‐based registered charity.

## Supporting information


**Data S1.** Supplementary Information.

## Data Availability

Data sharing is not applicable to this article as findings were derived from published literature and no new data were generated or analysed.
