# Supplementary material for: Drug use among people in prison: A global review of epidemiology, harms and interventions
Source: Addiction. 2025 Nov 20;121(4):749–64. doi: 10.1111/add.70245 (PMC12980310; doi:10.1111/add.70245)
Supplement: Supplementary file 1 — Data S1. Supplementary Information. [file ADD-121-749-s001.docx]

### Key publications informing our literature review

#### Review articles

Austin A, Favril L, Craft S, Thliveri P, Freeman TP. Factors associated with drug use in prison: a systematic review of quantitative and qualitative evidence. Int J Drug Policy. 2023;122:104248.

Baranyi G, Fazel S, Langerfeldt SD, Mundt AP. The prevalence of comorbid serious mental illnesses and substance use disorders in prison populations: a systematic review and meta-analysis. Lancet Public Health. 2022;7(6):e557-e68.

Baranyi G, Scholl C, Fazel S, Patel V, Priebe S, Mundt AP. Severe mental illness and substance use disorders in prisoners in low-income and middle-income countries: a systematic review and meta-analysis of prevalence studies. Lancet Glob Health. 2019;7(4):e461-e71.

Borschmann R, Keen C, Spittal MJ, Preen D, Pirkis J, Larney S, et al. Rates and causes of death after release from incarceration among 1 471 526 people in eight high-income and middle-income countries: an individual participant data meta-analysis. Lancet. 2024;403(10438):1779-88.

Carpentier C, Royuela L, Montanari L, Davis P. The global epidemiology of drug use in prison. In: Kinner SA, Rich JD, editors. Drug use in prisoners. Oxford: Oxford University Press; 2018. p. 17-41.

de Andrade D, Ritchie J, Rowlands M, Mann E, Hides L. Substance use and recidivism outcomes for prison-based drug and alcohol interventions. Epidemiol Rev. 2018;40(1):121-33.

Doyle MF, Shakeshaft A, Guthrie J, Snijder M, Butler T. A systematic review of evaluations of prison-based alcohol and other drug use behavioural treatment for men. Aust N Z J Public Health. 2019;43(2):120-30.

Edwards L, Jamieson SK, Bowman J, Chang S, Newton J, Sullivan E. A systematic review of post-release programs for women exiting prison with substance-use disorders: assessing current programs and weighing the evidence. Health Justice. 2022;10:1.

Favril L, Rich JD, Hard J, Fazel S. Mental and physical health morbidity among people in prisons: an umbrella review. Lancet Public Health. 2024;9(4):e250-e60.

Favril L, Shaw J, Fazel S. Prevalence and risk factors for suicide attempts in prison. Clin Psychol Rev. 2022;97:102190.

Fazel S, Yoon IA, Hayes AJ. Substance use disorders in prisoners: an updated systematic review and meta-regression analysis in recently incarcerated men and women. Addiction. 2017;112(10):1725-39.

Grella CE, Ostile E, Scott CK, Dennis M, Carnavale J. A scoping review of barriers and facilitators to implementation of medications for treatment of opioid use disorder within the criminal justice system. Int J Drug Policy. 2020;81:102768.

Grella CE, Ostlie E, Watson DP, Scott CK, Carnevale J, Dennis ML. Scoping review of interventions to link individuals to substance use services at discharge from jail. J Subst Abuse Treat. 2022;138:108718.

Horton M, McDonald R, Green TC, Nielsen S, Strang J, Degenhardt L, et al. A mapping review of take-home naloxone for people released from correctional settings. Int J Drug Policy. 2017;46:7-16.

Kendall S, Redshaw S, Ward S, Wayland S, Sullivan E. Systematic review of qualitative evaluations of reentry programs addressing problematic drug use and mental health disorders amongst people transitioning from prison to communities. Health Justice. 2018;6(1):4.

Kronfli N, Bromberg DJ, Wolff H, Montanari L, Vasyliev S, Altice FL. Improving implementation of needle and syringe programmes to expand, scale up, and sustain evidence-based prevention interventions for HIV and hepatitis C in prisons. Lancet Public Health. 2025;10(1):e63-e70.

Larney S, Stoové M, Kinner SA. Substance use after release from prison. In: Kinner SA, Rich JD, editors. Drug use in prisoners. Oxford: Oxford University Press; 2018. p. 85-98.

Lazarus JV, Safreed-Harmon K, Hetherington KL, Bromberg DJ, Ocampo D, Graf N, et al. Health outcomes for clients of needle and syringe programs in prisons. Epidemiol Rev. 2018;40(1):96-104.

Macdonald C, Macpherson G, Leppan O, Tran LT, Cunningham EB, Hajarizadeh B, et al. Interventions to reduce harms related to drug use among people who experience incarceration: systematic review and meta-analysis. Lancet Public Health. 2024;9(9):e684-e99.

Malta M, Varatharajan T, Russell C, Pang M, Bonato S, Fischer B. Opioid-related treatment, interventions, and outcomes among incarcerated persons: a systematic review. PLoS Med. 2019;16(12): e1003002.

Merrall ELC, Kariminia A, Binswanger IA, Hobbs MS, Farrell M, Marsden J, et al. Meta-analysis of drug-related deaths soon after release from prison. Addiction. 2010;105(9):1545-54.

Mitchell O, Wilson DB, Eggers A, MacKenzie DL. Assessing the effectiveness of drug courts on recidivism: a meta-analytic review of traditional and non-traditional drug courts. J Crim Just. 2012;40(1):60-71.

Moazen B, Dolan K, Moghaddam SS, Lotfizadeh M, Duke K, Neuhann F, et al. Availability, accessibility, and coverage of needle and syringe programs in prisons in the European Union. Epidemiol Rev. 2020;42(1):19-26.

Moore KE, Hacker RL, Oberleitner L, McKee SA. Reentry interventions that address substance use: a systematic review. Psychol Serv. 2020;17(1):93-101.

Moore KE, Roberts W, Reid HH, Smith KMZ, Oberleitner LMS, McKee SA. Effectiveness of medication assisted treatment for opioid use in prison and jail settings: a meta-analysis and systematic review. J Subst Abuse Treat. 2019;99:32-43.

Mundt AP, Baranyi G, Gabrysch C, Fazel S. Substance use during imprisonment in low- and middle-income countries. Epidemiol Rev. 2018;40(1):70-81.

Norman C. A global review of prison drug smuggling routes and trends in the usage of drugs in prisons. WIREs Forensic Sci. 2023;5(2):e1473.

Russell C, George TP, Chopra N, Le Foll B, Matheson FI, Rehm J, et al. Feasibility and effectiveness of extended-release buprenorphine (XR-BUP) among correctional populations: a systematic review. Am J Drug Alcohol Abuse. 2024;50(5):567-86.

Small W, McNeil R. Understanding the risk environment surrounding drug use in prisons: the unique contributions of qualitative research. In: Kinner SA, Rich JD, editors. Drug use in prisoners. Oxford: Oxford University Press; 2018. p. 161-74.

Treitler P, DiGioia-Laird V, Long B. Peer support services for individuals with health-related needs reentering the community after incarceration: a scoping review of program elements and outcomes. Health Justice. 2025;13(1):51.

van de Baan FC, Montanari L, Royuela L, Lemmens PHHM. Prevalence of illicit drug use before imprisonment in Europe: results from a comprehensive literature review. Drug Educ Prev Policy. 2022;29(1):1-12.

Varsaneux O, Charest M, Ma K, Stone J, Brouwers M, Kronfli N, et al. Identifying barriers and facilitators to accessing harm reduction services in prisons: a systematic narrative synthesis. Int J Drug Policy. 2025;143:104761.

Wirtz AL, Yeh PT, Flath NL, Beyrer C, Dolan K. HIV and viral hepatitis among imprisoned key populations. Epidemiol Rev. 2018;40(1):12-26.

Woods A, Foley C, Conigrave KM, Asare-Doku W, Shakeshaft A, Settumba-Stolk S, et al. Extended-release pharmacotherapies for substance use disorders in incarcerated populations: a systematic review. Addiction. 2025;120(5):835-59.

#### Primary studies

Andersen SN, Hyatt JM, Lobmaier P, Stavseth MR, Bukten A. Leaving their drugs at the gate? Exploring changes in drug use from before to during incarceration in Norway. Int J Offender Ther Comp Criminol. 2025;69(9):1207-29.

Balawajder EF, Ducharme L, Taylor BG, Lamuda PA, Kolak M, Friedmann PD, et al. Factors associated with the availability of medications for opioid use disorder in US jails. JAMA Netw Open. 2024;7(9):e2434704.

Bernard CL, Rao IJ, Robison KK, Brandeau ML. Health outcomes and cost-effectiveness of diversion programs for low-level drug offenders: a model-based analysis. PLoS Med. 2020;17(10):e1003239.

Binswanger IA, Nowels C, Corsi KF, Long J, Booth RE, Kutner J, et al. Return to drug use and overdose after release from prison: a qualitative study. Addict Sci Clin Pract. 2011;32(1):57-8.

Bronson J, Berzofsky M. Indicators of mental health problems reported by prisoners and jail inmates, 2011–12. Washington: Bureau of Justice Statistics; 2017.

Bukten A, Lund IO, Kinner SA, Rognli EB, Havnes IA, Muller AE, et al. Factors associated with drug use in prison – results from the Norwegian offender mental health and addiction (NorMA) study. Health Justice. 2020;8(1):10.

Bukten A, Virtanen S, Hesse M, Thylstrup B, Kvamme TL, Seid AK, et al. The prevalence of substance use disorders among people in Norwegian, Danish and Swedish prisons: a multi-national cohort study, 2010-19. Addiction. 2024;119(7):1264-75.

Chang Z, Larsson H, Lichtenstein P, Fazel S. Psychiatric disorders and violent reoffending: a national cohort study of convicted prisoners in Sweden. Lancet Psychiatry. 2015;2(10):891-900.

Chang Z, Lichtenstein P, Larsson H, Fazel S. Substance use disorders, psychiatric disorders, and mortality after release from prison: a nationwide longitudinal cohort study. Lancet Psychiatry. 2015;2(5):422-30.

Cumming C, Kinner SA, McKetin R, Young JT, Li I, Preen DB. The predictive validity of the Alcohol, Smoking and Substance Involvement Screening Test (ASSIST) for moderate- to high-risk cannabis, methamphetamine and opioid use after release from prison. Addiction. 2023;118(6):1107-15.

Cunningham EB, Hajarizadeh B, Amin J, Bretana N, Dore GJ, Degenhardt L, et al. Longitudinal injecting risk behaviours among people with a history of injecting drug use in an Australian prison setting: the HITS-p study. Int J Drug Policy. 2018;54:18-25.

Favril L. Drug use before and during imprisonment: drivers of continuation. Int J Drug Policy. 2023;115:104027.

Fovet T, Wathelet M, Benbouriche M, Benradia I, Roelandt JL, Thomas P, et al. Substance use, substance use disorders, and co-occurring psychiatric disorders in recently incarcerated men: a comparison with the general population. Eur Addict Res. 2022;28(5):368-76.

Houdroge F, Colledge-Frisby S, Kronfli N, Winter RJ, Carson J, Stoove M, et al. The costs and benefits of a prison needle and syringe program in Australia, 2025-30: a modelling study. Med J Aust. 2025;222(8):396-402.

Houdroge F, Kronfli N, Stoove M, Scott N. Cost-benefit analysis of Canada's prison needle exchange program for the prevention of hepatitis C and injection-related infections. CMAJ. 2024;196(43):1401-12.

Lokdam NT, Stavseth MR, Skjaervo I, Bukten A. Treatment utilization among people with drug use disorders in prison: a national longitudinal cohort study. Health Justice. 2024;12(1):46.

Lokdam NT, Stavseth MR, Bukten A. Drug use and re-imprisonment: a prospective study of the Norwegian Offender Mental Health and Addiction (NorMA) cohort. Drug Alcohol Depend Rep. 2022;5:100127.

Maruschak LM, Bronson J, Alper M. Alcohol and drug use and treatment reported by prisoners. Washington: Bureau of Justice Statistics; 2021.

Montanari L, Royuela L, Mazzilli S, Vandam L, Alvarez E, Llorens N, et al. Prevalence of drug use before and during imprisonment in seven European countries (2014–2018). J Community Psychol. 2024;52(8):1015-30.

Pape H, Lobmaier P, Bukten A. An evaluation of eight short versions of the Drug Use Disorder Identification Test (DUDIT). A prison population study. Drug Alcohol Depend Rep. 2022;3:100043.

Virtanen S, Aaltonen M, Latvala A, Forsman M, Lichtenstein P, Chang Z. Effectiveness of substance use disorder treatment as an alternative to imprisonment. BMC Psychiatry. 2024;24(1):260.

Winter RJ, Stoove M, Agius PA, Hellard ME, Kinner SA. Injecting drug use is an independent risk factor for reincarceration after release from prison: a prospective cohort study. Drug Alcohol Rev. 2019;38(3):254-63.
